# Non-operative treatment versus appendectomy for acute uncomplicated appendicitis: A randomized controlled trial

**DOI:** 10.12669/pjms.37.5.4016

**Published:** 2021

**Authors:** Muhammad Nadeem Sajjad, Fatima Naumeri, Sehrish Hina

**Affiliations:** 1 Dr. Muhammad Nadeem Sajjad, MBBS. Department of Pediatric Surgery, King Edward Medical University/Mayo Hospital, Lahore, Pakistan; 2 Dr. Fatima Naumeri, MCPS, FCPS. Department of Pediatric Surgery, King Edward Medical University/Mayo Hospital, Lahore, Pakistan; 3 Dr. Sehrish Hina, MBBS. Department of East Surgery, King Edward Medical University/Mayo Hospital, Lahore, Pakistan

**Keywords:** Acute appendicitis, Children, Non-operative treatment, Conservative management, Uncomplicated appendicitis, Non-perforated appendicitis, Appendectomy

## Abstract

**Objectives:**

To compare the outcome of non-operative treatment (NOT) with antibiotics versus surgical management of uncomplicated appendicitis in children.

**Methods:**

This randomized clinical trial (NCT 04030741) was carried out in Pediatric Surgery Department, Mayo Hospital from September 2018 to September 2019. Total sample size was 180, and after informed consent patients were randomly allocated in two groups. All children between 5 and 15 years of age and having pediatric appendicitis score (PAS) >7 were included. Patients with previous abdominal surgery, peritonitis, appendicular mass, or intra-abdominal abscess were excluded. Children in NOT group (Group A) were given intravenous Meropenem and Metronidazole for 48 hours and after starting oral, antibiotics were continued orally for 7 days. In Group B, children underwent appendectomy. Failure of NOT was labeled if there was abscess formation or peri-appendiceal fluid collection on ultrasound, development of peritonitis or recurrence of appendicitis.

**Results:**

In Group A, mean age was 9.56±1.8 years and in Group B 10.11±1.8 years. There were 123 male and 57 female patients. Group B had 100% successful outcome. NOT (Group A) had successful outcome in 75 patients (83.3%) and failure was noted in 15 (16.7%). Five needed operation within 48 hours, all had appendicolith, and 10 patients presented within 6 months. Raised total leukocyte count (p value < 0.0001) and C reactive protein (p value < 0.04) levels were noted with failure of NOT.

**Conclusions:**

In this study, success of NOT was 84% so a trial of NOT in cases of uncomplicated appendicitis should be considered in children.

## INTRODUCTION

Acute Appendicitis has an estimated lifetime risk of about 6.7-8.6%.^1^,^2^ Though the peak incidence is in second decade, overall, 11.4% children present in the emergency with acute appendicitis.^3^

The gold standard treatment for appendicitis is considered to be appendectomy.^4^ Appendectomy is an invasive procedure which leads to disturbance in child’s daily routine activities.^5^ Reported complication rates of this curative procedure are from 5% - 10%, with serious complications occurring in 1% to 7% of patients.^2^,^3^,^6^

Non-operative treatment (NOT) with wait-and-see approach and medical treatment, reserving appendectomy for complicated appendicitis only, decreases the hospital stay and cost of treatment.^6^ NOT leads to regression lymphoid follicles which subsequently decrease inflammation and obstruction.^7^ With medical management, it is possible to avoid surgery and its complications thus decreasing the morbidity due to acute appendicitis in children. Still to establish efficacy of NOT in children further randomized controlled trials (RCT) are needed.^8^,^9^

Sonia Maita and co-authors in a recent meta-analysis reported success rate of NOT as 92% after analyzing the published literature. They also reported that 16% later on needed appendectomy due to recurrent appendicitis.^9^ Huang L et al reported 90.5% success rate of NOT for acute uncomplicated appendicitis in a previous meta-analysis.^10^ Bachur and co-authors analyzed data of various United States’ hospitals retrospectively and revealed that NOT was associated with more emergency visits and hospitalization, and ultimately 46% had appendectomy in one year follow up, thus necessitating the need for further multi-centric RCT.^11^ Even with the need for readmission due to recurrent appendicitis in some children, overall cost was less in cases of NOT and it is taken as a cost effective modality with low hospital burden.^6^

The objective of this study was to compare the outcome of NOT versus appendectomy for uncomplicated appendicitis in children. Secondarily we wanted to evaluate the factors which led to failure of conservative management in non-perforated appendicitis. The rationale was that no local clinical trial is available to establish the efficacy of non-operative treatment with antibiotics in children, and only few trials are done internationally.^8^-^10^

## METHODS

This randomized clinical trial (NCT 04030741, IRB 201/RC/KEMU, dated: 12-03-2018) was carried out in Pediatric Surgery department, Mayo Hospital from September 2018 to September 2019. A sample size of 180 (90 in each group) was calculated at 5% level of significance and 80% power of test and taking expected frequency of success in NOT group is 89.6% and operative treatment is 100%.^6^ Sampling technique was non probability purposive sampling.

All children between 5 and 15 years of age of both genders admitted in the pediatric surgery emergency and having pediatric appendicitis score (PAS ≥ 7) were included. Patients with previous history of abdominal surgery, previous admission for NOT, clinically having peritonitis, appendicular mass, on ultrasound having abscess formation, complex peri-appendiceal fluid, or appendicolith, and/or raised C-reactive protein (CRP) were excluded.

After informed consent from parents/guardians, all patients inducted in the study were randomly divided into two groups: Non-operative treatment (Group A) and operative treatment (Group B), using computer generated number. Each patient was evaluated and relevant data according to the predesigned pro-forma was collected and documented. Age, duration of symptoms, body temperature, C-reactive protein (CRP), total leukocyte count (TLC), neutrophil concentrations, Ultrasound (USG) findings and PAS score were noted at the time of admission. Children in non-operative treatment group were given intravenous meropenem (10 mg/kg/dose intravenous infusion 8 hourly) and metronidazole (20 mg/kg/day intravenous divided doses 8 hourly) for at least 48 hours. Once the child started tolerating oral intake and clinically improved, the treatment was changed to oral ciprofloxacin (10 mg/kg/dose twice daily) and metronidazole (20 mg/kg/day two divided doses) for another 8 days. Group B received 3 doses of antibiotics and underwent open appendectomy through modified McBurney’s incision and muscle splitting. Rutherford Morrison extension with muscle cutting was done only for cases of complicated appendicitis. In cases of perforation only, intravenous antibiotics were continued for 5 days. Supportive care was given equally to all the patients as per protocol of treatment with regular vital monitoring. Improvement or development of complications was noted.

Failure of non-operative treatment was defined if any one of the following is seen: abscess formation or complex peri-appendiceal fluid collection seen on ultrasonography, the need for surgery (due to worsening of symptoms evaluated by history, physical examination and repeat USG) within 48 hours, or recurrence of appendicitis within 6 months.

Patients of both groups were discharged when started tolerating a light diet, and were afebrile for 24 hours, mobile, and had adequate pain relief on oral analgesics. Follow up duration was 6 months. Figure explains the clinical flow of this RCT.

**Fig.1:**
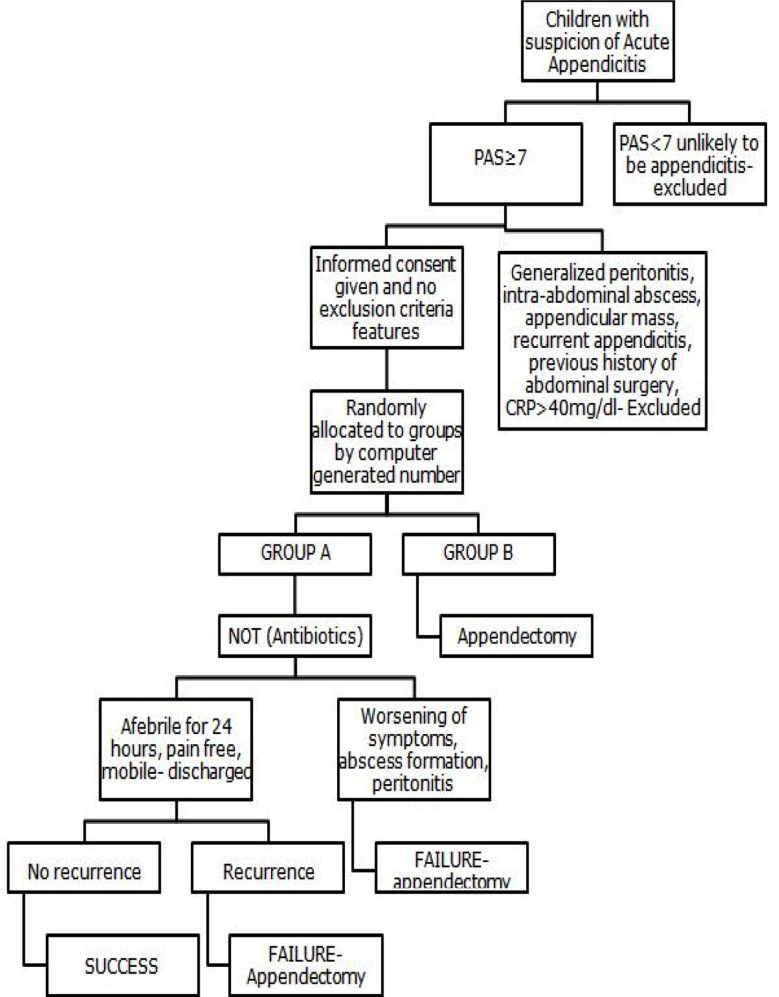
Flow Chart of randomized controlled trial.

The data was analyzed using SPSS version 23. Mean and standard deviation were calculated for quantitative variables like age, weight, temperature, TLC, neutrophils, PAS, and CRP. Frequency and percentages were calculated for qualitative variables like gender, and success/failure. Chi square test was used to compare the efficacy of two treatment modalities. P value of < 0.05 was taken as statistically significant. Effect modifiers like age, gender, duration of symptoms were controlled by stratification. Post stratification chi square test/ t test was applied. Mean difference of quantitative variables in were compared by t test. P value <0.05 was taken as statistically significant. Logistic regression was used to determine the association between age, gender, duration of symptoms, PAS scores, TLC, CRP levels and failure/success of NOT.

## RESULTS

Total patients enrolled were 180. There were 123 (68.3%) boys and 57 (31.7%) girls in the study. In Group A (n=90), there were 65 (72.2%) boys and 25 (27.8%) girls. In Group B (n=90), there were 58 (64.4%) boys and 32 (35.6%) girls. Mean and standard deviation of variables (age, weight, duration of symptoms, temperature, TLC, Neutrophils, CRP, and PAS) of both groups and all enrolled patients is given in Table-I.

**Table-I:** Mean and standard deviation of baseline variables.

Variables	Group		Total (n=180)

	A (n=90)	B (n=90)	
Age (years)	9.56±1.82	10.11±1.83	9.84±1.84
Duration of symptoms (hours)	21.68±10.6	18.98± 11.82	20.33±11.27
Temperature (^0^F)	99.84±1.29	99.87±1.28	99.86±1.28
TLC (10^9^/l) *	11311.11±2696.83	11322.22±2697.6	11316.67±2689.68
Neutrophil (cells/µl)	63.87±13.06	63.53±13.02	63.7±13.01
CRP (mg/dl)*	7.77±1.8	7.79±1.76	7.78±1.75
PAS *	7.74±0.9	7.79±0.92	7.77±0.89
Weight (kg)	17.52+4.62	18.47+5.11	17.78+4.89

* TLC (Total leukocyte count), CRP (C-reactive protein), PAS (Pediatric Appendicitis Score).

Comparison of the success of non-operative treatment (NOT) with antibiotics and operative treatment for uncomplicated appendicitis in children showed that success was recorded in 83.3% (n=75) in Group-A, whereas 100% (n=90) in Group-B (p value 0.0001). Failure of NOT was seen in 15 (16.7%) patients. Five needed appendectomy within 48 hours and all (100%) had appendicolith, while 10 patients presented within 6 months. None of these 10 patients had perforation or appendicolith.

For effect modifiers, stratification revealed that NOT was successful in 54 (n=65) boys and 21 (n=25) girls. NOT failure was noted in 1 out of 9 children between age 5-6 years (p value 0.5), in 3 out of 26 children between age 7-9 years (p value 0.08), and in 11 out of 55 children between age 10-15 years (p value 0.001).

Failure of conservative treatment with regards to duration of symptoms showed that only one out of 19 patients (p value 0.18) had failure if presented in less than 12hour, whereas 8 out of 57 patients had failure of NOT between 12-24hour (p value 0.01) and 6 out of 14 patients had failure of treatment when presented after 24 hours (p value 0.007).

Logistic regression revealed no association or correlation between age, gender, duration of symptom, PAS scores and failure or success of NOT. Mean difference of TLC and CRP was statistically significant in failure or success of NOT, as can be seen in Table-II.

**Table-II:** Comparison of quantitative variables in successful or failed non-operative treatment (Group A).

Variables	Success of not	Mean ± Standard deviation	P Value
Age (years)	Yes (n=75)	9.46 ± 1.81	0.24
	No (n=15)	10.07 ± 1.83	
Duration of symptoms (hours)	Yes (n=75)	20.83 ± 10.66	0.15
	No (n=15)	25.13 ± 9.08	
Total leukocyte count (10^9^/l)	Yes (n=75)	10760 ± 2347.2	0.000005
	No (n=15)	14066.7±2711.5	
C reactive protein (mg/dl)	Yes (n=75)	7.6 ± 1.8	0.04
	No (n=15)	8.6 ± 1.24	
Pediatric Appendicitis Score	Yes (n=75)	7.67 ± 0.76	0.58
	No (n=15)	7.8 ± 1.15	

## DISCUSSION

Non Operative Treatment avoids negative appendectomy and operation related complications in children,^2^ and this RCT established successful conservative management of uncomplicated appendicitis in around 84% children. Fifteen patients had failure of NOT. Five underwent appendectomy within 48 hours due to increasing tenderness. Histopathology was suggestive of acute appendicitis in all cases, and appendicolith was present in all cases. Ten patients presented with recurrent appendicitis within 6 months of follow up and histopathology revealed that all had appendicitis, though no appendicolith. In Group B, 100% successful outcome was noted (p value 0.0001). Seven (7.8%) patients had perforated or gangrenous appendix. One patient (1.1%) presented with adhesion obstruction in 6 months follow up.

The only published pilot RCT conducted by Svensson JF and colleagues randomized 50 patients (26 patients underwent appendectomy and 24 conservatively managed) and reported initial failure in 2 (8.3%) patients, though later on 6 more patients underwent appendectomy in conservative group. They reported that in recurrent appendicitis patients, histopathology was positive in one patient only, so labeling success of NOT as around 92%.^12^ In one prospective study, out of 197 children with uncomplicated appendicitis, 82 (42%) needed appendectomy within 48 hours and only 2.5% developed perforation, while 115 (58%) children didn’t require any surgical intervention.^13^ In one multi-centric analysis of over 4000 children, failure of NOT was high and in patients initially managed with NOT, 46% needed appendectomy in a year, and 14% had perforations.^11^ Success rate of non-operative treatment is 94.6% at the time of discharge, 89.2% at 30 days and 75.7% after 1 year as reported by Minneci PC et al.^6^ Similarly another study reported success of NOT as 75%.^14^ A locally published prospective study in adults reported failure of NOT in around 21 (22%) patients over a year.^15^

The systematic review done by Gorter and colleagues reported that around 62-81% children didn’t need appendectomy.^16^ Meta-analysis by Maita et al suggested 16% would need appendectomy, despite initial success of 92%.^9^ And another meta-analysis reported initial success in 97%, though 14% had recurrent appendicitis on follow-up.^8^ All these meta-analysis focus on the need of further RCTs’ and long follow-up. To our knowledge, this is second RCT being published.

Failure or success of NOT depends on different factors. One of these factors is selection criteria. PAS score 7, shorter duration of symptoms, no appendicolith or complex peri-appendiceal fluid on ultrasound led to success of NOT. Intra-luminal fluid and/or appendicolith on ultrasound was associated with higher failure rates.^17^ Patients with signs of perforation/ sepsis should not be considered for NOT as established in a prospective study.^13^ Other studies showed that children younger than 6 years and/or presenting after 48 hours are less likely to be managed conservatively.^11^,^14^ Literature also suggest that initial failure of conservative management was associated with appendicolith,^3^,^10^,^11^ this was also noted in our study.

Additional factors along with appendicolith presence, causing failure of non operative management include raised CRP, marked leukocytosis (more than 15,000 WBC/µl), bandemia, and extensive disease on CT scans as reported by Howell EC and co-authors.^18^ In our study, we also established that CRP and raised TLC were significantly associated with failure of conservative management.

In this study, there was male preponderance (68.3%) and older children (mean age 9.8±1.8 years) with 55 patients in Group A and 61 in Group B over 9 years old. This also reported in literature and a local study published that 77.1% boys presented with acute appendicitis.^19^

The use of PAS scores for indicating appendicitis in children has been validated by different studies. Recently Parveen and colleagues validated PAS scores by evaluating ultrasound findings and histopathology report.^20^ Another study also documented that PAS≥7 was strongly indicative of appendicitis and reported rate of negative appendectomy as 0%.^21^ A local study showed that Alvarado score of 6 or more has a diagnostic accuracy of 82.9% in predicting acute appendicitis in children.^22^ Still in this RCT we have used criteria of PAS≥7 for enrolling patients.

The concept of NOT is dependent on patients’ family willingness to revisit hospital in case of recurrence.^6^ In this RCT we only selected patients residing in Lahore, as patients visiting from periphery or already having difficult access to the hospital, can’t be offered NOT ethically. Other aspect to consider is prolonged use of antibiotics and its’ cost. Although available free of cost in public sector hospitals across Pakistan, still abroad as well cost of NOT is considered less than surgical management of appendicitis.^6^ Though long-term analysis of NOT in terms of cost and antibiotics resistance is still unavailable.^8^,^9^

Another important aspect to consider is possibility of failure of NOT in long term follow-up. Five years follow-up of children revealed that 46% underwent appendectomy, and 14% had histopathological confirmed diagnosis of recurrent appendicitis.^23^ Similar rates of 14%^8^ and 16%^9^ recurrence have been reported previously as well. Despite chances of recurrence, NOT should be offered in selected children as preservation of appendix helps in enhancing immunity. Quiet a few studies have shown that appendix is a source of pluripotent cells with a potential to help in gut repair and help in recolonization of normal commensals after diarrhea.^24^,^25^

Main limitation of this study is being single centered and having shorter follow up. Also, all histopathology reports of appendectomies were not available as few patients were lost to follow up.

## CONCLUSION

In this study, success of NOT was 84% so a trial of NOT in cases of uncomplicated appendicitis should be considered in children. Marked leukocytosis and/or C-reactive proteins levels, and presence of appendicolith led to failure of conservative treatment.

### Authors’ Contribution:

**MNS:** Designed, data collection, statistical analysis, and final approval of manuscript.

**FN:** Did manuscript writing, editing of manuscript, final approval and accountable for manuscript.

**SH:** Data entry, review of manuscript, and final approval of manuscript.
